# The Multidimensional Pathogenesis of Susceptibility to Depression in Sjögren’s Syndrome: An Integrated Approach from Immune–Inflammatory to Psychosocial Factors

**DOI:** 10.3390/ijms272110038

**Published:** 2026-11-09

**Authors:** Qiumei He, Xiaoqiao Gu, Qianqian Yang, Niqin Xiao, Xi Liu, Kuanmeng Chi, Zhaohu Xie, Zhaofu Li

**Affiliations:** First School of Clinical Medicine, Yunnan University of Chinese Medicine, Kunming 510499, China; 15108897926@163.com (Q.H.); g15908883654@163.com (X.G.); qqy0802@126.com (Q.Y.); xnq2022@126.com (N.X.); 15812147568@163.com (X.L.); 15308700826@163.com (K.C.)

**Keywords:** Sjögren’s syndrome, depression, inflammation, psychosocial factors

## Abstract

Sjögren’s syndrome is a chronic systemic autoimmune disease with primary (pSS) and secondary forms. Epidemiological evidence suggests a higher prevalence of depressive symptoms and depression in pSS than in the general population, indicating possible disease-specific susceptibility. The underlying mechanisms remain unclear, limiting early identification and targeted intervention. This narrative review examines five domains of depression susceptibility in pSS: inflammatory pathways, neuroendocrine regulation, the gut–brain axis, medication effects, and psychosocial factors. Interleukin-6, tumor necrosis factor-α, and interferon-α may contribute by activating the indoleamine 2,3-dioxygenase–kynurenine pathway, downregulating brain-derived neurotrophic factor, and inhibiting hippocampal neurogenesis. Hypothalamic–pituitary–adrenal axis dysfunction may provide a neuroendocrine link, whereas gut microbial imbalance may affect emotional regulation through immune and neural signaling. Glucocorticoids, hydroxychloroquine, and calcineurin inhibitors may increase neuropsychiatric risk through multiple pathways. Somatic symptoms and social stress may reinforce a psychosocial vicious cycle. Much of the mechanistic evidence comes from major depressive disorder, animal models, or in vitro studies; causality has not been established in pSS. The proposed multidimensional model is hypothetical and offers a framework for early screening and targeted intervention in pSS with depression.

## 1. Introduction

Sjögren’s syndrome (SS) is a chronic inflammatory connective tissue disease characterized by exocrine gland damage, primarily affecting the salivary and lacrimal glands. Clinical manifestations include dry mouth and dry eyes. Immune-mediated inflammation can also damage the lungs, kidneys, nervous system, skin, thyroid, stomach, liver, joints, and muscles. SS is the second most common connective tissue disease after rheumatoid arthritis [1]. It is classified as primary SS (pSS) or secondary SS (sSS), depending on its association with other connective tissue diseases. pSS predominantly affects middle-aged and older women, particularly during perimenopause and postmenopause. The peak age at onset is approximately 56 years [2]. Its prevalence in China ranges from 0.29% to 0.77% [3].

Clinical research has increasingly examined mental health in patients with pSS. Several cohort studies report a significantly higher risk of depression in pSS than in rheumatoid arthritis or systemic lupus erythematosus [4]. Depression is the most common psychiatric comorbidity in pSS. Its reported prevalence varies, although the overall findings are generally consistent. A Chinese cross-sectional study reported a prevalence of 36.9% [5]. Using the Hamilton Depression Rating Scale (HAMD) and Beck Depression Inventory (BDI), Koçer et al. reported a comorbidity rate of 40.60% [6].

The definition of “depression” varies across studies. Screening and assessment tools include the Patient Health Questionnaire-9 (PHQ-9), Hospital Anxiety and Depression Scale (HADS), BDI, and HAMD. Some studies classify depression using scale-specific thresholds, whereas psychiatrists apply diagnostic criteria for major depressive disorder (MDD) [7]. A positive screen indicates possible depressive symptoms and is not equivalent to an MDD diagnosis. These tools differ in purpose, items, cutoff scores, and suitability for specific populations. Reported prevalence differences may therefore partly reflect assessment methods and diagnostic thresholds rather than true differences in prevalence [8]. Pain, fatigue, and somnolence affect quality of life in pSS, with depression acting as a key moderating factor [9]. Salai et al. identified depression and cognitive impairment as the most common psychological complications, alongside sleep disorders, anxiety, and other problems [10]. Women aged 40–60 years constitute a high-risk group for depression in pSS. Many are menopausal or perimenopausal, and estrogen fluctuations may be an important trigger [11]. Depression risk is highest during the first and fifth years after pSS diagnosis [12]. Compared with healthy controls, patients with pSS have higher rates of depression and anxiety, more characteristic symptoms, and greater symptom severity [5]. Patients with both pSS and depression report more clinical symptoms, most commonly fatigue [13]. Sleep disturbances and fatigue are also frequently reported manifestations of this comorbidity [14].

Treatment of pSS with depression follows a tiered approach. Pharmacotherapy typically involves adding antidepressants to standard pSS treatment, which has limited efficacy against depression and fatigue. Anxiolytics or antidepressants, such as paroxetine, are therefore considered reasonable adjunctive options [15]. However, meta-analyses associate second-generation and atypical antidepressants with increased dry mouth risk [16]. Second-generation agents include selective serotonin reuptake inhibitors (SSRIs), serotonin–norepinephrine reuptake inhibitors (SNRIs), and other types. Biologics, including rituximab and belimumab, improve pSS disease activity and are important options for refractory disease with systemic symptoms. Evidence remains insufficient to determine their effects on depression or cognitive function in pSS [17,18]. Traditional Chinese medicine (TCM) uses syndrome differentiation to guide treatment based on “nourishing yin fluids, moistening dryness, and regulating emotions.” Combining classical TCM formulas or acupuncture with Western medications has produced significant therapeutic effects. Although TCM has shown distinctive clinical benefits, its guidelines remain incomplete, procedures lack standardization, and mechanisms remain poorly understood. Current approaches consequently address symptoms rather than underlying causes, reflecting uncertainty about the mechanisms linking pSS and depression susceptibility. A multidimensional case-based analysis of this comorbidity’s pathogenesis is therefore needed.

## 2. Literature Search and Evidence Synthesis

This narrative review is not a systematic review or meta-analysis. We searched PubMed, Web of Science, and the China National Knowledge Infrastructure (CNKI) from database inception to August 23, 2026. Searches combined subject headings and free-text terms covering pSS, depression or anxiety, and the mechanistic domains examined in this review. Representative English and Chinese Boolean search strings were as follows. English: ((“Sjögren’s syndrome” OR “primary Sjögren’s syndrome” OR pSS) AND (depression OR depressive symptoms OR anxiety)) AND (inflammation OR cytokines OR “HPA axis” OR “gut microbiota” OR glucocorticoid OR hydroxychloroquine OR “calcineurin inhibitor” OR psychosocial). Chinese: (干燥综合征 OR 原发性干燥综合征) AND (抑郁 OR 焦虑) AND (炎症 OR 细胞因子 OR 下丘脑-垂体-肾上腺轴 OR 肠道菌群 OR 糖皮质激素 OR 羟氯喹 OR 钙调神经磷酸酶抑制剂 OR 心理社会). Eligible publications included studies, reviews, guidelines, animal experiments, in vitro studies, and case reports. These addressed pSS/SS with depression or anxiety, inflammatory pathways, neuroendocrine factors, the gut–brain axis, drug-related psychiatric adverse effects, or psychosocial factors. We excluded duplicates, publications without accessible full texts, irrelevant studies, and abstracts with insufficient information. Two researchers independently screened titles and abstracts before reviewing full texts. Disagreements were resolved by consensus or consultation with a third researcher. Heterogeneity in designs, populations, outcomes, and mechanisms precluded quantitative meta-analysis. Evidence was stratified by source: pSS clinical studies, general depression studies, animal studies, in vitro studies, and case reports/conference abstracts. Records were not systematically counted because this was a narrative review. Table 1 summarizes the main limitations.

## 3. Disease-Related Susceptibility to Depression: Inflammatory, Neurotransmitter, and Endocrine Factors

### 3.1. Abnormal Immune Function and Inflammation in pSS and Depression

#### 3.1.1. Immunological Basis of pSS: Dysregulation of Innate and Adaptive Immunity

The immunological mechanisms of pSS primarily involve dysregulated innate and adaptive immunity. Innate abnormalities include impaired dendritic cell antigen presentation, macrophage infiltration, epithelial barrier defects, and inflammasome activation. Dendritic cells and dysfunctional salivary gland epithelial cells share antigen-presenting functions. Pathological epithelial cells release abundant chemokines and adhesion molecules that stimulate inflammasomes, initiating innate immune responses. These responses recruit dendritic cells, lymphocytes, and macrophages to the glands, disrupting immune tolerance. Interferon (IFN) upregulation subsequently increases B-cell activating factor (BAFF) expression in epithelial cells, dendritic cells, and T cells, activating adaptive immunity. The resulting B-cell proliferation, subset dysregulation, and modulation of autoantibody levels ultimately impair glandular secretion and may produce extraglandular manifestations [19]. Adaptive abnormalities include B-cell subset imbalances (BAFF/cytokine imbalance) and T-cell dysfunction. Affected subsets include type 1, type 2, and type 17 T helper cells (Th1, Th2, and Th17), regulatory T (Treg) cells, and T follicular helper (Tfh) cells. Th1 cells and their products are major drivers of chronic tissue damage. These cells directly damage exocrine glands by secreting inflammatory molecules, including IFN-γ, interleukin-17 (IL-17), and interleukin-21 (IL-21). They may also indirectly promote autoantibody production through B-cell interactions [20]. Th17 cells are major salivary gland infiltrates and release IL-17, which induces inflammation, chemokine release, and lymphocyte recruitment, activation, and migration. IL-17 also enhances abnormal autoimmunity by promoting B- and T-cell survival and inhibiting T-cell apoptosis, contributing to glandular damage [21]. Studies have confirmed abundant FOXP3+ Treg cells in pSS exocrine gland tissues. However, a shift in the Th17/Treg balance toward the proinflammatory Th17 axis further worsens the disease [22,23]. Tfh cells also contribute to lymphoid follicle and ectopic germinal center formation in pSS salivary glands [24]. B-cell hyperactivation is a key pathogenic mechanism, mediating ectopic germinal center-like structures and autoantibody production in exocrine glands. Circulating CD27^+^ memory B cells are reduced in pSS but accumulate significantly in the salivary glands. Through positive feedback, B cells promote CD4^+^ T-cell proliferation and development, accelerating disease progression [19]. SS is characterized by persistently elevated Th17 cells and associated proinflammatory cytokines in salivary gland tissue and serum. These include interleukin-6 (IL-6), IL-17, and interleukin-12 (IL-12) [25]. Tumor necrosis factor-α (TNF-α) and interleukin-1β (IL-1β) also have significant roles in pSS pathogenesis [26,27,28,29].

#### 3.1.2. Immunological Basis of Depression: Cytokine Signaling and Neuroimmune Interactions

Patients with depression show abnormal expression of inflammatory mediators and cytokines. Peripheral levels of IL-1β, IL-6, TNF-α, C-C motif chemokine ligand 2 (CCL2), and C-reactive protein (CRP) are elevated. Genetic variations in these proinflammatory cytokines are associated with depression severity [82,83]. Cytokines such as interleukin-1α (IL-1α), TNF-α, and IL-6 can downregulate brain-derived neurotrophic factor (BDNF), inhibit hippocampal neurogenesis, and promote depression [84,85]. IL-1 and TNF-α can activate p38 mitogen-activated protein kinase (MAPK) signaling, increasing serotonin reuptake pump expression and function. This reduces synaptic serotonin utilization in experimental animals and triggers depression [86]. Cytokines also disrupt tetrahydrobiopterin (BH4) and phenylalanine metabolism, both of which are associated with depressive symptoms [30,87]. The proinflammatory cytokine IL-6 has a key role in immune responses to inflammatory diseases [31,32]. By regulating T-cell function, IL-6 promotes T-cell proliferation and infiltration into the central nervous system (CNS). This affects brain function and neurotransmitter production, ultimately leading to anxiety and depression [33]. A cross-sectional study of 60 women with pSS found significantly higher IL-6 concentrations in patients with depression than in those without depression (*p* < 0.001). IL-6 independently predicted depression in pSS (odds ratio [OR] = 3.23, 95% confidence interval [CI]: 1.07–9.80, *p* = 0.038) [32]. We therefore hypothesize that IL-6 contributes importantly to the onset and progression of pSS with anxiety and depression. A meta-analysis of 36 studies found significantly elevated IL-17A levels in patients with depression, alongside a trend toward higher Th17 cell counts. In pSS, Th17-derived IL-17 promotes chemokine release, lymphocyte recruitment, and inflammatory amplification. These mediators may cross the blood–brain barrier (BBB) and affect central emotional regulation networks [34]. Patients with systemic inflammatory diseases have an increased risk of depression [88]. Chronic local inflammation damages the BBB, allowing peripheral cytokines to enter the brain parenchyma. These cytokines activate brain immune cells, including microglia, or exacerbate their responses. Subsequent changes include neuroendocrine dysfunction, abnormal neurotransmitter metabolism, and structural and functional alterations in emotion-related regions, such as the prefrontal cortex, hippocampus, and amygdala. These changes ultimately trigger depression [89,90]. Dantzé et al. proposed indoleamine 2,3-dioxygenase (IDO) as a key enzyme linking peripheral inflammation to central depressive-like behaviors. Interferon-α (IFN-α) and TNF-α can activate IDO, redirecting tryptophan metabolism toward the tryptophan mononucleotide pathway. This reduces the availability of the serotonin precursor and generates neurotoxic metabolites, such as quinolinic acid, preventing tryptophan from contributing to CNS serotonin synthesis [35,36]. Resulting hippocampal dysfunction causes depression, cognitive slowing, and other cognitive impairments [91]. The affected glial cells, including microglia and astrocytes, have reduced glutamate reuptake capacity, enhancing glutamatergic signaling. Alongside inhibited serotonin synthesis, this may trigger pain perception and depressive behaviors; the ensuing cascade may further exacerbate inflammation [10]. Imbalances in kynurenine pathway metabolites may contribute to inflammation-related depression [36,92]. These mechanisms lack direct clinical validation in pSS. Nevertheless, chronic inflammation in pSS suggests a possible role for the IDO–kynurenine pathway in depression susceptibility. Peripheral cytokines do not enter the brain solely through passive BBB disruption. Cytokines, including IL-1α, IL-1β, TNF-α, and IL-6, can cross into the brain through saturable receptor-mediated transport systems on BBB endothelial cells [93]. Such transport conveys peripheral inflammatory signals without compromising barrier integrity. The BBB also differs structurally across brain regions. Peri-cortical organs (CVOs), including terminal plate vessels, subcortical structures, and the last area of the medulla, have fenestrated capillary endothelia. These lack typical BBB tight junctions, increasing permeability to peripheral cytokines, hormones, and peptides. CVOs exhibit substantial regional differences in vascular permeability and tight junction protein expression compared with typical BBB regions [94,95]. Peripheral low-molecular-weight inflammatory mediators may therefore enter specific neural regions more readily, triggering localized rather than uniformly distributed neuroimmune responses. Resident brain immune cells can also produce cytokines in response to peripheral low-molecular-weight stimuli, amplifying and sustaining central inflammation [96]. Peripheral stimulation and subsequent autonomous cytokine production by brain cells can affect neurons and glia. By inhibiting serotonin synthesis, enhancing glutamatergic signaling, and disrupting neuroendocrine function, this cascade contributes to depression and cognitive impairment [97].

### 3.2. HPA Axis Dysfunction as a Candidate Neuroendocrine Link

#### 3.2.1. Chronic Stress and HPA Axis Homeostasis in SS

The hypothalamic–pituitary–adrenal (HPA) axis comprises the hypothalamus, pituitary gland, and adrenal gland. It regulates stress responses, emotional regulation, and energy metabolism through cascades involving corticotropin-releasing hormone (CRH), adrenocorticotropic hormone (ACTH), and cortisol. Some patients with SS have significantly lower basal ACTH and cortisol levels than healthy individuals [37]. Valtysdóttir et al. found intact cortisol synthesis in women with pSS but lower serum dehydroepiandrosterone sulfate (DHEA-S) levels and a higher cortisol/DHEA-S ratio [38].

Cortisol levels rise continuously under chronic stress. Persistent dryness, pain, and fatigue predispose patients with SS to chronic stress, during which initially elevated cortisol levels may persist. Using salivary cortisol to assess HPA axis function, Glavina et al. found significantly higher concentrations in pSS than in controls. Depression and anxiety scores were respectively 4 and 6 times those of controls, suggesting short-term HPA axis hyperactivity [39]. These apparently conflicting findings may reflect dynamic HPA axis dysregulation and differences between basal activity and stress responses. Early disease or initial chronic stress may reduce HPA axis activity through compensatory regulation. Persistent inflammation and stress may subsequently shift the axis toward excessive activation. Prolonged cortisol elevation suppresses hypothalamic CRH and pituitary ACTH secretion, weakening HPA axis negative feedback and causing hormonal imbalance [40].

#### 3.2.2. Depression-Related HPA Axis Hyperactivity and a Vicious Cycle

The HPA axis is a major neuroendocrine regulator involved in depression pathogenesis. Its hyperactivity and elevated CRH levels are major contributors to depression and anxiety. Prolonged exposure to pathogens, stress, and other factors can increase HPA axis activity and central CRH secretion. Elevated peripheral ACTH and cortisol then trigger psychiatric disorders such as depression [98].

HPA axis dysfunction may be a central link between pSS and depression. Basal HPA axis function is reduced in pSS, with lower CRH and cortisol levels and a weaker response to CRH stimulation. Under chronic stress, cortisol levels remain elevated. Prolonged cortisol exposure suppresses hypothalamic CRH and pituitary ACTH secretion, weakening HPA axis negative feedback. Depression, in contrast, typically involves HPA axis hyperactivity and impaired negative feedback under chronic stress [40]. Their combined effects reduce anti-inflammatory inhibition, increasing proinflammatory cytokine release, including IL-6 and TNF-α. This exacerbates inflammation of exocrine glands outside the SS and activates the kynurenine pathway across the BBB. The resulting serotonin depletion and hippocampal neuronal damage trigger depressive behaviors [99,100]. pSS-related fatigue, sleep disturbances, and autonomic dysfunction are also closely associated with abnormal HPA axis circadian rhythms, further worsening mood. pSS and depression may thus reinforce each other through a neuroimmune–endocrine vicious cycle, with HPA axis dysregulation as a key pathophysiological mechanism [101]. However, most evidence is indirect, and causality has not been established. We therefore consider HPA axis dysfunction a candidate neuroendocrine link between pSS and depression. Its direction, whether reduced or increased activity, and the distinction between basal activity and stress responses require clarification. Future studies should measure both basal HPA axis activity and dynamic stress responses, directly comparing patients with pSS with and without depression.

### 3.3. Gut–Brain Axis Dysregulation Linking Immune and Neural Function

#### 3.3.1. Gut Microbiota Dysregulation in SS

The gut microbiota comprises microorganisms normally present in the intestinal tract, predominantly Bacteroidetes, Firmicutes, Actinobacteria, and Proteobacteria. Bacteroidetes and Firmicutes account for 90% of the community [102]. Dysbiosis is common in autoimmune diseases, and microbiota changes contribute importantly to SS onset and progression. Population studies and animal experiments associate the degree of dysbiosis with SS severity [103]. SS onset may involve increased pathogenic bacteria and reduced commensal bacteria. Greater microbial diversity supports a more stable balance; reduced diversity allows pathogens to damage the mucosal barrier and promote inflammatory mediator release, disrupting immune homeostasis [104]. In pSS, bacteria associated with intestinal health, including *Bacteroides* and *Bifidobacterium*, decrease under chronic inflammation and may contribute to disease pathogenesis [105]. Deppavi et al. found higher fecal levels of *Arcobacter*, *Escherichia*, and *Streptococcus*, but lower levels of *Bifidobacterium* and fecal bacteria in pSS [103]. Increased *Escherichia* may promote inflammatory mediator release, autoimmunity, and autoimmune salivary gland inflammation, accompanied by elevated serum immunoglobulin G (IgG). Reduced butyrate-producing bacteria are also associated with pSS onset and progression [106]. Patients typically show increased Bacteroidetes, decreased Actinobacteria, and lower levels of the *Bifidobacterium* family, distinguishing them from healthy individuals [107]. Patients with pSS and dry eye show similar dysbiosis patterns, with the proportion of Bacteroidetes showing the most significant change [108,109]. Microbiota changes can also affect immune cells in ocular lymphoid tissues and reduce Treg cell numbers [110].

#### 3.3.2. Gut–Brain Axis Imbalance as a Contributor to Depression

Cytokines include proinflammatory mediators, such as IL-8 and TNF-α, and anti-inflammatory mediators, such as IL-10 and transforming growth factor-β (TGF-β). Chronic stress increases proinflammatory and decreases anti-inflammatory cytokine secretion, causing oxidative and nitrosative brain damage. IDO pathway activation then reduces tryptophan and serotonin availability, contributing to depression [111]. Cytokines also disrupt dopamine, glutamate, and serotonin metabolism, causing glutamate imbalance and excitotoxicity and reducing BDNF production, potentially leading to depressive symptoms [112]. The gut microbiota interacts with the intestinal mucosa to regulate the balance between proinflammatory and anti-inflammatory mediators. Proinflammatory dominance may trigger inflammation and depressive-like behaviors [105]. Chronic stress can damage the intestinal barrier, which comprises mucins and antimicrobial peptides, promoting dysbiosis and harmful bacterial expansion. These bacteria release toxins that stimulate intestinal epithelial cells and activate or overactivate the HPA axis, causing neuroendocrine dysfunction and depression [112]. Monoamine neurotransmitter deficiency, including reduced serotonin, norepinephrine, and dopamine, is the main cause of depression. Gut microbes can synthesize serotonin and melatonin, which affect the CNS through the vagus nerve. Dysbiosis reduces monoamine production, thereby triggering depression [113]. Gut–brain axis imbalance alone cannot fully explain depression; it may contribute through immune, endocrine, and neural pathways.

#### 3.3.3. pSS-Related Cerebral Microvascular and White Matter Lesions in Depression

The depression hypothesis identifies neuronal and nerve fiber damage and neurotransmitter receptor dysfunction as major causes of depression. Implicated regions include the frontal and temporal lobes and thalamus [114]. Microvascular and ischemic lesions in specific brain regions can impair function in pSS [115]. Zhong Jingmei et al. found higher rates of ischemic and hemorrhagic cerebrovascular disease in these regions in pSS than in controls. This suggests that pSS-related depression may involve inflammation-mediated cerebral vascular damage [116,117]. CNS white matter lesions (WMLs) are associated with depression and are more common in pSS. Greater WML burden in pSS may also be associated with depression [118]. Patients with pSS and depression may have weaker somatosensory cortical functional connectivity and microstructural alterations in the corticospinal tract and major white matter tracts [119]. Serum antibodies against the N-methyl-D-aspartate receptor (NMDAR) NR2 subtype are higher in patients with pSS and depression than in those without depression [120]. Antibody positivity is also associated with hippocampal atrophy, leading to cognitive impairment and emotional symptoms [121].

#### 3.3.4. An Integrated Gut–Brain Axis Model: From Association to Hypothesis

Patients with pSS exhibit significant dysbiosis, with fewer beneficial bacteria, including butyrate producers, and more proinflammatory bacteria. This disrupts intestinal immune balance, including the Treg/Th17 balance, promoting autoimmunity [41]. These changes can trigger peripheral and central inflammation; central inflammation directly affects emotional regulation pathways, ultimately producing depressive- and anxiety-like behaviors [42]. Patients with pSS commonly experience anxiety, depression, and other emotional disturbances. General depression studies, studies in other disease populations, and animal experiments show that microbial metabolites, such as short-chain fatty acids, can directly affect emotions through the gut–brain axis [43]. Dysbiosis can disrupt HPA axis function, altering circadian rhythms and increasing basal stress hormone levels, including cortisol. Sustained HPA axis overactivation may lead to anxiety and depressive symptoms [122]. Evidence of altered microbial composition comes from observational studies in pSS populations and animal models. These studies establish an association between dysbiosis and pSS severity, but do not directly establish that dysbiosis causes depressive symptoms in pSS [Figure 1].

**Figure 1:**
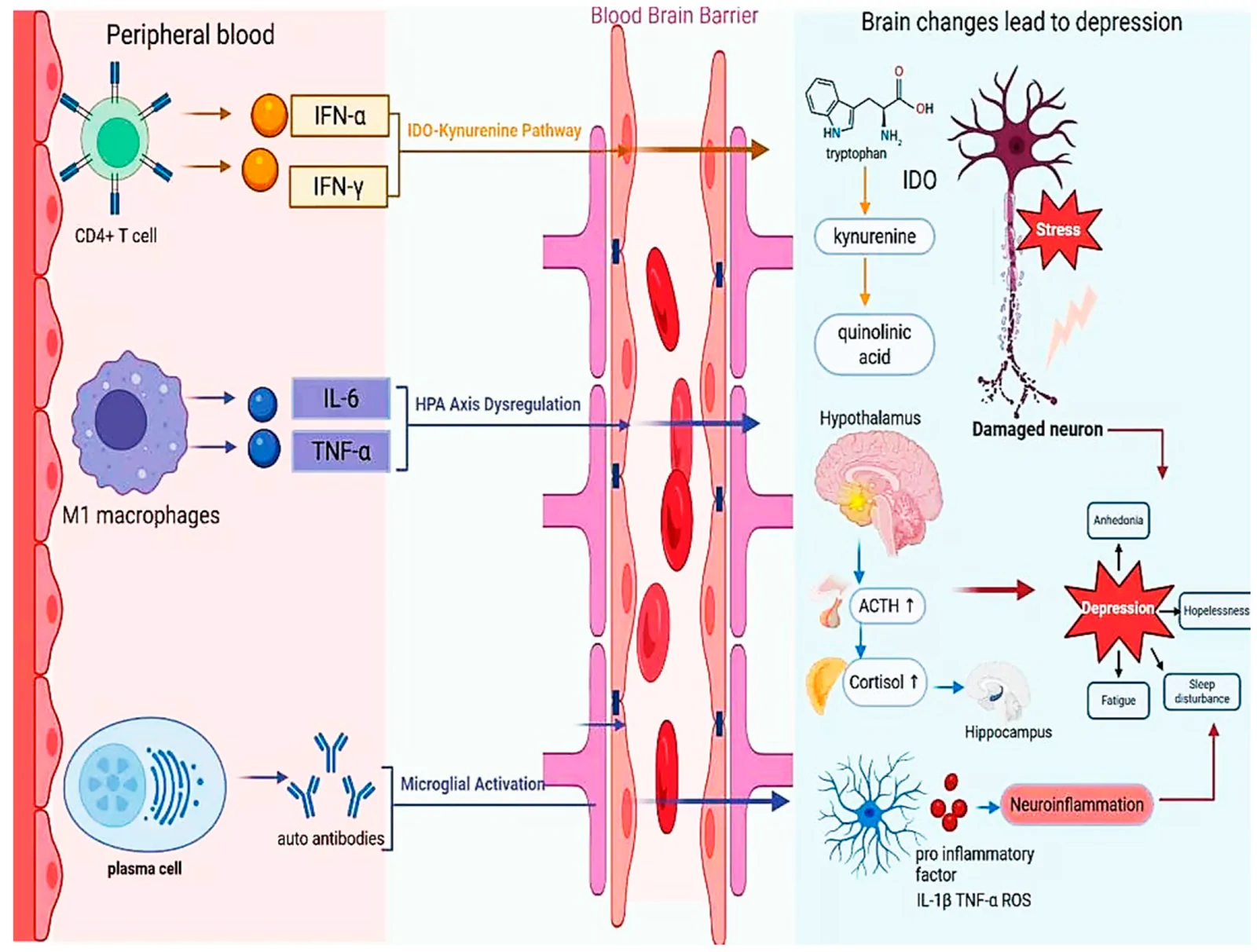
Possible molecular links between peripheral inflammation in pSS and depression through neuroimmune–endocrine pathways. pSS-related peripheral inflammation crosses the BBB through three major pathways, driving central inflammation and depressive-like behaviors. (1) IDO–kynurenine pathway: IFN-α/IFN-γ activate IDO, shifting tryptophan metabolism toward kynurenine, reducing 5-hydroxytryptamine (5-HT) synthesis, and producing neurotoxic quinolinic acid that damages neurons. (2) HPA axis pathway: proinflammatory mediators, including IL-6 and TNF-α, activate the HPA axis. Adrenocorticotropic hormone-releasing hormone (CRH), ACTH, and cortisol progressively increase, damaging hippocampal structure and function. (3) Microglial activation pathway: peripheral inflammatory mediators cross the BBB and activate brain microglia. These release IL-1β, TNF-α, and reactive oxygen species (ROS), amplifying CNS inflammation. The three pathways converge in the hippocampus and other emotion-related regions, manifesting as anhedonia, helplessness, fatigue, and sleep disturbances. The figure integrates pSS, general depression, animal, and in vitro evidence. Some pathways lack direct validation in patients with both pSS and depression, making this a hypothetical framework. This figure is the author’s original work.

## 4. Iatrogenic Depression Risk and the Dual Effects of Medications

### 4.1. Glucocorticoids and Multifactorial Impairment of Neuroplasticity

#### 4.1.1. Glucocorticoid Use in pSS and Depression Risk

Glucocorticoids (GCs) are not standard first-line therapy for pSS, and evidence does not support their efficacy for its primary symptom, dryness. They may, however, form part of initial treatment for specific organ involvement, including neurological, renal, or hematologic disease. Such involvement is indicated by a high EULAR Sjögren’s syndrome disease activity index (ESSDAI) score [44]. GCs can have substantial neurological adverse effects, including depression, mania, anxiety, and cognitive impairment. Associations with depressive and manic episodes are particularly notable [45]. Clinical studies, case reports, pharmacovigilance data, and animal studies have confirmed the depressogenic effects of GCs. Adult rats treated with dexamethasone exhibit significant depression-like behavior [46]. Large multicenter clinical trials report depression rates as high as 26% in patients with systemic autoimmune diseases receiving GCs. Depression scores nearly doubled from pretreatment levels, a statistically significant difference (*p* < 0.001). High doses and a psychiatric history independently predict depression in these patients [47,48]. Treatment-related psychiatric disturbances most commonly emerge 3–4 days after initiation but can occur at any time during therapy. New or recurrent episodes may also develop after discontinuation, and depression is more frequent in women [49]. Clinicians must therefore monitor mental status throughout GC therapy, including initiation, long-term maintenance, tapering, and discontinuation.

#### 4.1.2. HPA Axis Hyperactivity and GR/MR-Mediated Impairment of Synaptic Plasticity

GCs trigger depression through several neurobiological pathways, with HPA axis hyperactivity and impaired negative feedback as key pathophysiological mechanisms [50]. GCs bind glucocorticoid receptors (GR) and mineralocorticoid receptors (MR), which are highly expressed in the brain. This disrupts synaptic plasticity in emotion-related regions, including the hippocampus and amygdala. GCs inhibit hippocampal CA1 long-term potentiation (LTP) and induce long-term depression (LTD), reducing synaptic transmission efficiency. They also modulate glutamate receptor expression, exacerbating synaptic dysfunction and ultimately disrupting emotion-related neural circuits [49,51].

#### 4.1.3. JNK-Mediated GR Ser246 Phosphorylation and BDNF Downregulation

Studies have confirmed that c-Jun N-terminal kinase (JNK) mediates GR hyperphosphorylation at serine 246, inhibiting GR transcriptional activity and promoting GC-induced depression. Inhibiting this pathway significantly alleviates neuropathic damage and abnormal behavioral phenotypes. These findings suggest that JNK-mediated GR Ser246 phosphorylation is a key mechanism of depression after GC treatment [52]. BDNF is a major regulator of neuroplasticity. High GC concentrations significantly reduce hippocampal BDNF expression, inhibit neuronal survival and synaptic remodeling, and induce depression-like pathological changes [53].

#### 4.1.4. NF-κB–NLRP3 Inflammasome Signaling, Microglial Activation, and Oxidative Stress

A reinforcing cycle of neuroinflammation and oxidative stress also contributes to pathogenesis. Patients with SS already have elevated peripheral IL-6 and TNF-α, which can cross the BBB and activate microglia [32]. GCs further activate microglia through the nuclear factor κB (NF-κB)–NLRP3 inflammasome pathway, inducing abundant IL-1β and IL-18 release and amplifying CNS inflammation [54]. NLRP3 also promotes microglial immune memory. Inflammation may therefore persist after GC stimulation ceases, leading to recurrent depression [55]. Prolonged GC exposure disrupts cerebral oxidative balance, causing mitochondrial dysfunction and ROS accumulation that can damage hippocampal neurons. ROS can further activate the NLRP3 inflammasome, reinforcing oxidative stress and neuroinflammation and exacerbating depression-related pathological damage [56,57].

#### 4.1.5. Monoamine Neurotransmitter System Dysregulation

Monoamine neurotransmitter system dysfunction is a key pathological feature of depression, and GCs can disrupt this system. By regulating organic cation transporter 3 (OCT3), they alter synaptic cleft levels of serotonin (5-HT), norepinephrine (NE), and dopamine (DA) [58]. GR functional changes can directly influence prefrontal monoamine oxidase A (MAO-A) expression. Dexamethasone treatment significantly increased Krüppel-like factor 11 (KLF11) and MAO-A expression in prefrontal cortical neurons [59]. MAO-A degrades monoamines, including serotonin, norepinephrine, and dopamine [60]. Excessive activity reduces their levels and can trigger depression [61]. High GC levels may also impair monoamine neurotransmission by inducing glutamate-mediated excitotoxicity [62].

### 4.2. Hydroxychloroquine, Serotonin Transport, and Neuronal Autophagy

Hydroxychloroquine (HCQ) is the most commonly used immunomodulator for pSS, but psychiatric adverse effects may occur during treatment. Evidence comes from case reports, pharmacovigilance data, and observational studies; causality remains uncertain. Reported symptoms include anxiety, depression, and psychosis. Concomitant fluoroquinolones or nonsteroidal anti-inflammatory drugs (NSAIDs) increase neuropsychiatric risk [63]. Symptoms may range from anxiety and insomnia to severe psychiatric disturbances [64]. Animal and in vitro studies suggest that HCQ may reduce hippocampal serotonin transporter (*SLC6A4*) expression. SLC6A4 regulates serotonin reuptake from the synaptic cleft, affecting synaptic serotonin concentrations and emotional regulation [65]. HCQ is lipid-soluble and crosses the BBB to enter the CNS. Its CNS concentration is 10 to 20 times that in peripheral tissues, providing a basis for neurotoxicity [66]. As a weak lysosomal alkalizing agent, HCQ can inhibit lysosome–autophagosome fusion, impairing abnormal protein clearance and maintenance of neuronal synaptic structure [67]. Persistent autophagy disruption may cause neuronal stress and dysfunction, indirectly leading to depression-like behavior.

### 4.3. Calcineurin Inhibitors and Synaptic Dysfunction as a Possible Contributor to Depression

Tacrolimus (Tac) and cyclosporine (CsA) are calcineurin inhibitors (CNIs). In pSS, they are primarily administered as topical eye drops for dry eye. Randomized controlled trials show improvements in pSS-related ocular surface symptoms [68].

Whether long-term CNI use increases depression risk remains unclear. Route, dose, systemic exposure, and treatment duration must be considered when assessing this risk. Several theoretical mechanisms have been proposed. First, inhibiting prefrontal calmodulin-dependent phosphatase activity disrupts mammalian target of rapamycin (mTOR) signaling homeostasis. This reduces synaptic plasticity and alters neurotransmission, ultimately triggering depression [69]. Second, chronic stress reduces calmodulin-dependent phosphatase activity in the hippocampal CA3 region. Injecting cyclosporine A into this region can directly induce depression-like behavior [70]. Third, this phosphatase interacts with the serotonin transporter C-terminal domain and regulates serotonin reuptake. Its endogenous activity has antidepressant effects, whereas long-term calcium channel blocker use disrupts this regulation and increases depression susceptibility [71]. Finally, cyclosporine may promote nitric oxide production in cerebral vascular endothelial cells and astrocytes, impairing BBB function and causing related neurotoxicity [72] [Figure 2].

**Figure 2:**
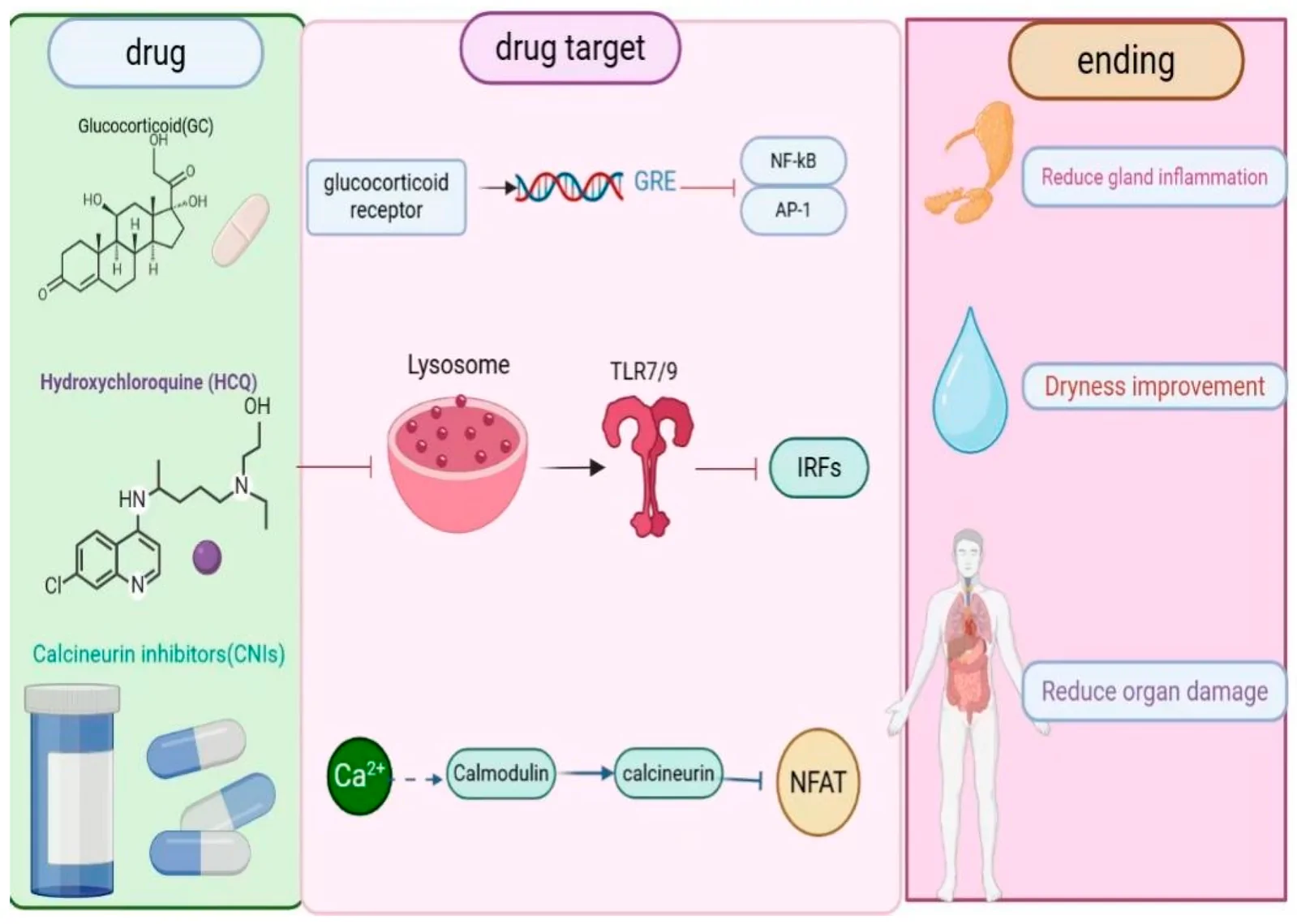
Molecular targets and clinical effects of medications commonly used for SS. Three medication classes commonly used for SS and their principal mechanisms are shown. (1) GCs bind GR and regulate transcription factors, including NF-κB, AP-1, and IRF, broadly suppressing inflammation. (2) HCQ, a weak lysosomal alkalinizing agent, inhibits plasmacytoid dendritic cell activation and autoantibody production by blocking Toll-like receptor 7/9 (TLR7/9) signaling. (3) CNIs, including tacrolimus and cyclosporine, inhibit calcineurin activity and prevent NF-AT nuclear translocation, suppressing T-cell activation and cytokine release. These immunomodulatory actions alleviate glandular inflammation and dryness symptoms and reduce systemic organ damage. Abbreviations: GR, glucocorticoid receptor; NF-κB, nuclear factor κB; AP-1, activator protein-1; IRFs, interferon regulatory factors; NF-AT, nuclear factor of activated T cells; TLR, Toll-like receptor. The figure depicts clinical benefits only; neuropsychiatric adverse reactions are listed in Table 1. This figure was created by the authors.

## 5. Psychosocial Factors: Physical Symptoms and External Stress

Persistent dry mouth, dry eyes, fatigue, and sleep disturbances impose a substantial psychological burden on patients with pSS [15,73]. Dry mouth and dry eyes are associated with increased risks of depression, anxiety, and sleep disorders [74,75]. Cross-sectional studies identify oral health and dysphagia as significant predictors of anxiety and depression in pSS [5]. Taste disturbances, a burning tongue, and halitosis can also affect mood and cause psychological distress [76]. González et al. found significantly higher depression incidence in patients with pSS and dry eye syndrome. Rates were higher still with ocular discomfort, including foreign body sensation, light sensitivity, and burning [77]. Segal et al. reported abnormal fatigue in 67% of patients with pSS; 41% of these patients also had depressive symptoms. Depression was closely associated with fatigue severity [78]. A Dutch study found that 46.1% of patients frequently experienced poor sleep quality, with significantly higher depression prevalence in this group [79]. Patients with pSS and depression have higher disease activity, erythrocyte sedimentation rate (ESR), and pain levels than those without depression [80]. Patients may also experience personality changes, become overly preoccupied with their illness, or obtain inaccurate medical information through unofficial channels. These factors increase their risk of depression, anxiety, and other psychological disorders [15]. Compared with patients with rheumatoid arthritis, those with pSS score significantly higher on neuroticism and lower on extraversion and openness. Personality traits may therefore also confer depression risk [81]. Yang et al. and Šeromková et al. found significantly more negative life events in patients with pSS and depression than in those without depression. These events involved family, school, and work. Treatment costs and insufficient family and social support also contribute importantly to anxiety and depression [15].

## 6. Clinical Intervention: From Reactive Treatment to Stratified Management

### 6.1. Recommendations from Existing Guidelines and Consensus Statements

Clinical management of pSS focuses on relieving dryness and controlling and delaying systemic damage [123]. Dryness is managed with artificial tears, saliva substitutes, secretagogues, and oral care. Common treatments for joint pain include NSAIDs, low-dose corticosteroids, and methotrexate [124]. Systemic involvement is typically treated with GCs and immunosuppressants. For neurological involvement, options include medium- to high-dose GCs, gamma globulin, cyclophosphamide, or rituximab [125].

### 6.2. Depression Screening and Psychiatric Evaluation

Depression screening should be incorporated into routine follow-up for patients with confirmed pSS, using periodic assessments such as the PHQ-9 [126]. A positive screen is not a diagnosis of clinical depression and requires further psychiatric evaluation.

### 6.3. Antidepressant Selection

Treatment of pSS with comorbid depression should address the underlying disease and include appropriate antidepressant use [5]. SSRIs are recommended, with selection individualized to depression severity, previous treatment response, baseline xerostomia and xerophthalmia, and concomitant medications. Dry mouth and dry eyes should be monitored regularly. Artificial tears, saliva substitutes, secretagogues, and oral care should be intensified as needed. Current regimens remain ineffective at alleviating the clinical symptoms of both pSS and depression. Further mechanistic research is needed to identify therapeutic targets and develop more effective, targeted medications.

### 6.4. Multidisciplinary and Individualized Care

A multidisciplinary care model involving rheumatology, psychiatry, psychotherapy, and nursing is recommended. Individuals positive for anti-SSA/SSB antibodies who are at risk of disease but have no dry eye symptoms should receive education that builds confidence in treatment. Lifestyle measures include increasing environmental humidity, drinking plenty of water, maintaining eye and skin hygiene, and limiting screen time [127,128]. pSS is a chronic, intractable autoimmune disease with recurrent flares that impose substantial psychological stress, potentially compromising quality of life. Psychological counseling should therefore accompany pharmacotherapy [129]. Psychotherapy for depression supports positive clinician–patient relationships and treatment confidence while helping patients identify and change negative thoughts and behavioral responses. This alleviates depression and anxiety and improves quality of life. Physical activity, music, and movies can promote relaxation and positive psychological changes [10]. Aerobic exercise can also improve pSS-related depression and fatigue [130].

## 7. Limitations

This narrative review is not a systematic review or meta-analysis. Although we searched PubMed, Web of Science, and CNKI, the protocol was not registered. Publication and language biases may therefore have led to relevant studies being omitted. Considerable heterogeneity in designs, populations, outcomes, and mechanisms precluded meta-analysis. Much of the evidence is associative, and causal links between inflammation, HPA axis dysfunction, dysbiosis, drug effects, and depression have not been established in pSS. Effects of medication dose, treatment duration, and concomitant drugs are also incompletely understood. These limitations should guide interpretation of the review’s conclusions.

## 8. Conclusions and Future Research Directions

pSS may be associated with increased depression susceptibility, although much of the evidence is indirect. Inflammatory pathways, HPA axis dysfunction, dysbiosis, medication effects, and psychosocial factors may interact, but causal relationships remain unclear. IFN-α, IL-6, TNF-α, and IL-1β may affect central emotional regulation through IDO–tryptamine pathways, HPA axis dysfunction, BDNF downregulation, and autonomic dysfunction. GCs may increase depression risk by affecting BDNF, microglial activation, oxidative stress, and monoamine neurotransmission, although pSS-specific evidence remains insufficient. Case reports and animal/in vitro studies associate HCQ and CNIs with neuropsychiatric adverse reactions; causality in pSS remains unclear. Fatigue, pain, dryness, negative coping styles, and reduced social support may exacerbate depressive symptoms. Relevant clinical studies are few and generally small, with particularly limited treatment research in pSS with depression. The absence of specific treatment guidelines or consensus statements leaves clinicians without clear guidance. Future studies should follow patients from before diagnosis, longitudinally monitoring immune and inflammatory markers, medication use, and emotional changes. A comprehensive multifactorial model should integrate immunological, pharmacological, and psychological mechanisms. Animal experiments and related approaches should clarify these mechanisms and provide evidence on causal links between chronic inflammation and depression. Clinical trials should collect extensive data to assess the efficacy and safety of immunomodulators and antidepressants in patients with pSS and depression. The role of adjunctive interventions, including psychological counseling, also requires clarification to support the gradual development of stratified clinical management. The proposed multidimensional model remains hypothetical and identifies research directions for early recognition, mechanistic investigation, and targeted intervention in pSS with depression.

## Figures and Tables

**Table 1:** Major Mechanisms, Types of Evidence, and Limitations of pSS with Comorbid Depression.

Mechanism	Key Findings	Direct Evidence in pSS with Depression?	Evidence Source/Study Type	Evidence Strength	Key Limitations	Representative Refs
Immune-inflammation (Th1/Th17)	pSS: Th1/Th17 activation, Th17/Treg imbalance, elevated IL-6, IL-17, TNF-α, IFN-α. Depression: elevated Th17, IL-17A, IL-6. pSS with depression: IL-6 independently predicts depression (OR = 3.23).	Partial. IL-6-depression association directly shown in pSS patients (*n* = 60). Th17-depression link shown in general depression, not yet directly in pSS.	pSS clinical studies; general depression meta-analyses; animal studies	Moderate for IL-6–depression association in pSS; indirect for Th17–depression link	Small sample size; no longitudinal data; Th17 not directly measured in pSS with depression	[19,20,21,22,23,24,25,26,27,28,29,30,31,32,33,34]
IDO–kynurenine pathway	Pro-inflammatory cytokines (IFN-α, IFN-γ, TNF-α) activate IDO, diverting tryptophan to kynurenine pathway → reduced 5-HT, increased neurotoxic quinolinic acid.	Indirect. IDO activation shown in IFN-positive pSS patients; depression link inferred from general depression and animal studies.	pSS observational studies (IDO); general depression and animal studies (depression link)	Indirect	Direct evidence of IDO–depression link in pSS patients lacking	[35,36]
HPA axis	Contradictory findings: some studies show lower basal ACTH/cortisol; a recent study shows higher salivary cortisol and 4× depression scores in pSS. Dynamic dysregulation proposed.	Indirect. No study directly compares ACTH/cortisol between pSS with vs. without depression.	pSS clinical studies (contradictory); general depression studies	Weak/contradictory	Small samples; different assessment methods; disease stage and medication not controlled	[37,38,39,40]
Gut–brain axis	pSS: dysbiosis (↓ butyrate-producing bacteria, ↑ pro-inflammatory bacteria); Treg/Th17 imbalance. Depression link: indirect evidence from general depression, animal models, other diseases.	Indirect. Dysbiosis–depression causality not established in pSS.	pSS observational studies; animal models; general depression studies	Preliminary/indirect	No interventional or longitudinal data in pSS; causality not established	[41,42,43]
Drug-induced (GCs)	GCs: via GR/MR, interfere with hippocampal synaptic plasticity, activate NF-κB–NLRP3, downregulate BDNF, disrupt monoamine system.	Indirect. Evidence from systemic autoimmune disease clinical studies, case reports, pharmacovigilance, animal experiments; not pSS-specific.	Clinical observations, case reports, pharmacovigilance, animal studies	Moderate for general risk; weak for pSS-specific	Dose, duration, route not fully controlled; confounding by disease activity	[44,45,46,47,48,49,50,51,52,53,54,55,56,57,58,59,60,61,62]
Drug-induced (HCQ)	HCQ: reduces hippocampal *SLC6A4*, inhibits autophagy; CNS concentration 10–20× peripheral.	Indirect. Evidence from case reports, pharmacovigilance, animal/in vitro studies.	Case reports, pharmacovigilance, animal/in vitro studies	Weak	Causality uncertain; no controlled studies in pSS	[63,64,65,66,67]
Drug-induced (CNIs)	Tacrolimus/ciclosporin: may disrupt mTOR signaling, reduce calcineurin activity in CA3, affect 5-HT reuptake.	Indirect. Evidence mainly from systemic administration, animal models, or in vitro studies; not from topical ocular use in pSS.	Animal/in vitro studies; RCTs for ocular efficacy only	Weak for depression risk	Route, dose, systemic exposure, duration not considered; evidence mainly from systemic administration	[68,69,70,71,72]
Psychosocial	Dryness, fatigue, pain, sleep disturbance, personality traits (higher neuroticism), negative life events, economic burden, lack of social support.	Direct. Cross-sectional studies in pSS patients show associations between these factors and depression.	pSS cross-sectional and questionnaire studies	Moderate for association	Cannot establish causality; cross-sectional design; confounding	[15,73,74,75,76,77,78,79,80,81]

Abbreviations: Th1, type 1 T helper cell; Th17, type 17 T helper cell; Treg, regulatory T cell; IL-6, interleukin-6; OR, odds ratio; IL-17, interleukin-17; TNF-α, tumor necrosis factor-α; IFN-α, interferon-α; pSS, primary Sjögren’s syndrome; 5-HT, 5-hydroxytryptamine; IDO, indoleamine 2,3-dioxygenase; HPA, hypothalamic–pituitary–adrenal; ACTH, adrenocorticotropic hormone; GCs, glucocorticoids; GR, glucocorticoid receptor; MR, mineralocorticoid receptor; NF-κB, nuclear factor κB; NLRP3, NOD-like receptor pyrogenic protein domain-related protein 3; BDNF, brain-derived neurotrophic factor; HCQ, hydroxychloroquine; *SLC6A4*, solute carrier family 6 member 4; mTOR, mammalian target of rapamycin; CA3, hippocampal CA3 region; RCT, randomized controlled trial. "↓" as "decrease" and "↑" as "increase.

## Data Availability

The original contributions presented in this study are included in the article. Further inquiries can be directed to the corresponding author(s).

## References

[1] Witas R., Peck A.B., Ambrus J.L., Nguyen C.Q. (2019). Sjogren’s Syndrome and TAM Receptors: A Possible Contribution to Disease Onset. J. Immunol. Res..

[2] Qin B.D., Wang J.Q., Yang Z.X., Yang M., Ma N., Huang F.L., Zhong R.Q. (2015). Epidemiology of primary Sjögren’s syndrome: A systematic review and meta-analysis. Ann. Rheum. Dis..

[3] Baldini C., Talarico R., Tzioufas A.G., Bombardieri S. (2012). Classification criteria for Sjogren’s syndrome: A critical review. J. Autoimmun..

[4] Beider S., Stephan M., Seeliger T., Skripuletz T., Witte T., Ernst D. (2025). A comparative cross-sectional study of psychological distress, fatigue, and physical activity in patients with rheumatoid arthritis, systemic lupus erythematosus, and Sjögren’s disease. Front. Med..

[5] Cui Y.F., Xia L., Li L., Zhao Q., Chen S.N., Gu Z.F. (2018). Anxiety and depression in primary Sjögren’s syndrome: A cross-sectional study. BMC Psychiatry.

[6] Koçer B., Tezcan M.E., Batur H.Z., Haznedaroglu S., Goker B., Irkeç C., Cetinkaya R. (2016). Cognition, depression, fatigue, and quality of life in primary Sjögren’s syndrome: Correlations. Brain Behav..

[7] Saltiel P.F., Silvershein D. (2015). Major depressive disorder: Mechanism-based prescribing for personalized medicine. Neuropsychiatr. Dis. Treat..

[8] Hitchon C.A., Zhang L., Peschken C.A., Lix L.M., Graff L.A., Fisk J.D., Patten S.B., Bolton J., Sareen J., El-Gabalawy R. (2020). Validity and Reliability of Screening Measures for Depression and Anxiety Disorders in Rheumatoid Arthritis. Arthritis Care Res..

[9] Costa T., Rushton S.P., Watson S., Ng W.F., Tiago C. (2023). UK primary Sjögren’s Syndrome Registry.Depression in Sjögren’s syndrome mediates the relationship between pain, fatigue, sleepiness, and overall quality of life. Rheumatol. Immunol. Res..

[10] Salehi M., Zamiri A., Kim J., Texeira C., Shah K., Gunturu S. (2024). Exploring the Psychiatric Manifestations of Primary Sjögren’s Syndrome: A Narrative Review. Int. J. Rheumatol..

[11] Saraux A., Pers J.O., Devauchelle-Pensec V. (2016). Treatment of primary Sjögren syndrome. Nat. Rev. Rheumatol..

[12] Shen C.-C., Yang A.C., Kuo B.I., Tsai S.-J. (2015). Risk of Psychiatric Disorders Following Primary Sjögren Syndrome: A Nationwide Population-based Retrospective Cohort Study. J. Rheumatol..

[13] Segal B., Thomas W., Rogers T., Leon J.M., Hughes P., Patel D., Patel K., Novitzke J., Rohrer M., Gopalakrishnan R. (2008). Prevalence, severity, and predictors of fatigue in subjects with primary Sjögren’s syndrome. Arthritis Rheum..

[14] Tastekin F., Karatas E., Aydin O., Zihni F.Y., Kabasakal Y., Barutcuoglu B., Karabulut G. (2024). How Do Exercise, Sleep and Depression Affect Disease Scores and Quality of Life in Primary Sjögren’s Syndrome Patients?. Int. J. Gen. Med..

[15] Tianwen H., Yanhong L., Yubin L., Zhou Y., Zhao Y., Liu Y. (2022). Research progress on the pathogenesis and quality of life of patients with primary Sjögren’s syndrome complicated by depression. Clin. Exp. Rheumatol..

[16] Cappetta K., Beyer C., Johnson J.A., Bloch M.H. (2018). Meta-analysis: Risk of dry mouth with second generation antidepressants. Prog. Neuro-Psychopharmacol. Biol. Psychiatry.

[17] Neagu A.E., Opriș-Belinski D., Baciu T., Daia-Iliescu S., Cobilinschi C., Saulescu I. (2026). Beyond Dryness: Mapping the Psychological and Cognitive Burden in Sjögren’s Disease—A Narrative Review. J. Clin. Med..

[18] Marinho A., Delgado Alves J., Fortuna J., Faria R., Almeida I., Alves G., Araújo Correia J., Campar A., Brandao M., Crespo J. (2023). Biological therapy in systemic lupus erythematosus, antiphospholipid syndrome, and Sjögren’s syndrome: Evidence- and practice-based guidance. Front. Immunol..

[19] Chivasso C., Sarrand J., Perret J., Delporte C., Soyfoo M.S. (2021). The Involvement of Innate and Adaptive Immunity in the Initi-ation and Perpetuation of Sjögren’s Syndrome. Int. J. Mol. Sci..

[20] Alunno A., Carubbi F., Bistoni O., Bartoloni E., Valentini V., Gerli R. (2017). Novel Therapeutic Strategies in Primary Sjögren’s Syndrome. Isr. Med. Assoc. J..

[21] Alunno A., Ibba-Manneschi L., Bistoni O., Rosa I., Caterbi S., Gerli R., Manetti M. (2016). Mobilization of lymphatic endothelial precursor cells and lymphatic neovascularization in primary Sjögren’s syndrome. J. Cell. Mol. Med..

[22] Haskett S., Ding J., Zhang W., Thai A., Cullen P., Xu S., Petersen B., Kuznetsov G., Jandreski L., Hamann S. (2016). Identification of Novel CD4+ T Cell Subsets in the Target Tissue of Sjögren’s Syndrome and Their Differential Regulation by the Lymphotoxin/LIGHT Signaling Axis. J. Immunol..

[23] Matsui K., Sano H. (2017). T Helper 17 Cells in Primary Sjögren’s Syndrome. J. Clin. Med..

[24] Sandhya P., Kurien B.T., Danda D., Scofield R.H. (2017). Update on Pathogenesis of Sjogren’s Syndrome. Curr. Rheumatol. Rev..

[25] Katsifis G.E., Rekka S., Moutsopoulos N.M., Pillemer S., Wahl S.M. (2009). Systemic and local interleukin-17 and linked cytokines associated with Sjögren’s syndrome immunopathogenesis. Am. J. Pathol..

[26] Bayetto K., Logan R.M. (2010). Sjögren’s syndrome: A review of aetiology, pathogenesis, diagnosis and management. Aust. Dent. J..

[27] Lin J.C., Pan K.L., Li C.F., Lee K.F., Lin K.Y., Lin K.M., Lin C.Y. (2023). Altered subgroups of regulatory T cells in patients with primary Sjögren’s syndrome. Heliyon.

[28] Schett G., Dayer J.-M., Manger B. (2016). Interleukin-1 function and role in rheumatic disease. Nat. Rev. Rheumatol..

[29] Zhang S., Qu J., Wang L., Li M., Xu D., Zhao Y., Zhang F., Zeng X. (2021). Activation of Toll-Like Receptor 7 Signaling Pathway in Primary Sjögren’s Syndrome-Associated Thrombocytopenia. Front. Immunol..

[30] Neurauter G., Schrocksnadel K., Scholl-Burgi S., Sperner-Unterweger B., Schubert C., Ledochowski M., Fuchs D. (2008). Chronic immune stimulation correlates with reduced phenylalanine turnover. Curr. Drug Metab..

[31] Grebenciucova E., VanHaerents S. (2023). Interleukin 6: At the interface of human health and disease. Front. Immunol..

[32] Mrsić F., Vukasović I., Kuna A.T., Davidović B.L., Markeljević J. (2025). Association of inflammatory markers with depression and anxiety in female patients with primary Sjögren’s syndrome. Biochem. Medica.

[33] Zadka Ł., Dzięgiel P., Kulus M., Olajossy M. (2017). Clinical Phenotype of Depression Affects Interleukin-6 Synthesis. J. Interferon Cytokine Res..

[34] Moulton C.D., Malys M., Hopkins C.W.P., Rokakis A.S., Young A.H., Powell N. (2025). Activation of the interleukin-23/Th17 axis in major depression: A systematic review and meta-analysis. Eur. Arch. Psychiatry Clin. Neurosci..

[35] Arnone D., Saraykar S., Salem H., Teixeira A.L., Dantzer R., Selvaraj S. (2018). Role of Kynurenine pathway and its metabolites in mood disorders: A systematic review and meta-analysis of clinical studies. Neurosci. Biobehav. Rev..

[36] Müller N., Schwarz M.J. (2007). The immune-mediated alteration of serotonin and glutamate: Towards an integrated view of depression. Mol. Psychiatry.

[37] Johnson E.O., Vlachoyiannopoulos P.G., Skopouli F.N., Tzioufas A.G., Moutsopoulos H.M. (1998). Hypofunction of the stress axis in Sjögren’s syndrome. J. Rheumatol..

[38] Valtysdóttir S.T., Wide L., Hällgren R. (2001). Low serum dehydroepiandrosterone sulfate in women with primary Sjögren’s syndrome as an isolated sign of impaired HPA axis function. J. Rheumatol..

[39] Glavina A., Zurak P., Martinovic D., Glavina A., Zurak P., Gotovac M., XSupe-Domic D., Lugović-Mihić L. (2024). Evaluation of the Hypothalamic-Pituitary-Adrenal Axis in Patients with Primary Sjögren’s Disease. Medicina.

[40] Skopouli F.N., Katsiougiannis S. (2018). How stress contributes to autoimmunity-lessons from Sjögren’s syndrome. FEBS Lett..

[41] Cano-Ortiz A., Laborda-Illanes A., Plaza-Andrades I., del Pozo A.M., Cuadrado A.V., de Mora M.R.C., Leiva-Gea I., Sanchez-Alcoholado L., Queipo-Ortuño M.I. (2020). Connection between the Gut Microbiome, Systemic Inflammation, Gut Permeability and FOXP3 Expression in Patients with Primary Sjögren’s Syndrome. Int. J. Mol. Sci..

[42] Zhu Y., Zhang K., Luo Z., Song Y., Wang X. (2025). Gut microbiota on anxiety and depression in primary Sjogren’s syndrome: A novel insight. Heliyon.

[43] Fox R.I. (2005). Sjögren’s syndrome. Lancet.

[44] Ramos-Casals M., Brito-Zerón P., Bombardieri S., Bootsma H., De Vita S., Dörner T., Fisher B.A., Gottenberg J.E., Hernandez-Molina G., Kocher A. (2020). EULAR recommendations for the management of Sjögren’s syndrome with topical and systemic therapies. Ann. Rheum. Dis..

[45] Koning A., van der Meulen M., Schaap D., Satoer D.D., Vinkers C.H., van Rossum E., van Furth W.R., Pereira A.M., Meijer O.C., Dekkers O.M. (2024). Neuropsychiatric Adverse Effects of Synthetic Glucocorticoids: A Systematic Review and Meta-Analysis. J. Clin. Endocrinol. Metab..

[46] Laaziz A., El Mostafi H., Elhessni A., Touil T., Doumar H., Mesfioui A. (2022). Chronic clomipramine treatment reverses depressogenic-like effects of a chronic treatment with dexamethasone in rats. IBRO Neurosci. Rep..

[47] Hamouda M.M., Abdel-Hassib M., Shabana H., Almarshad F., Mohammed O.S., AYoub H.S., Ullah H., Hamed H.I., Alnabawy S.M., Abdel-Samiee M. (2025). Corticosteroid-induced mood changes in systemic autoimmune diseases: A multicentric study. Gastroenterol. Rev..

[48] Lagerberg T., Gustafsson T.T., Molero Y., Forton J., Sariaslan A., Chang Z., Larsson H., Lichtenstein P., Fazel S. (2026). Oral glucocorticoids and risk of psychiatric and suicidal behaviour outcomes: Population-based cohort study. Br. J. Psychiatry.

[49] Costa A.P., Mousinho M., Pires A.M., Simões G., Gomes A.I., Coelho A.R. (2025). From Mood Swings to Psychosis: Exploring the Psychiatric Side Effects of Corticosteroids. Eur. Psychiatry.

[50] Göver T., Slezak M. (2024). Targeting glucocorticoid receptor signaling pathway for treatment of stress-related brain disorders. Pharmacol. Rep..

[51] Chen F., Li L., Huang M., Wang Y., Wang L., Jin F., Yang L., Gao M., Li L., Wang Y. (2024). Natural product Kaji-ichigoside F1 exhibits rapid antidepression via activating the AMPA–BDNF–mTOR pathway and inhibiting the NMDAR–CaMKIIα pathway. Phytomedicine.

[52] Zhang J., Lin W., Tang M., Zhao Y., Zhang K., Wang X., Li Y. (2020). Inhibition of JNK ameliorates depressive-like behaviors and reduces the activation of pro-inflammatory cytokines and the phosphorylation of glucocorticoid receptors at serine 246 induced by neuroinflammation. Psychoneuroendocrinology.

[53] Duman R.S., Aghajanian G.K. (2012). Synaptic Dysfunction in Depression: Potential Therapeutic Targets. Science.

[54] Feng X., Zhao Y., Yang T., Song M., Wang C., Yao Y., Fan H. (2019). Glucocorticoid-Driven NLRP3 Inflammasome Activation in Hippocampal Microglia Mediates Chronic Stress-Induced Depressive-Like Behaviors. Front. Mol. Neurosci..

[55] Xu G., Yuan M., He H., Yi J., Li X., Yan H., Liu G., Wu Z., Zhang X., Chen Y. (2026). NLRP3-mediated trained immunity of microglia is involved in the recurrence-like episode of depressive disorders. Mol. Psychiatry.

[56] Correia A.S., Cardoso A., Vale N. (2023). Oxidative Stress in Depression: The Link with the Stress Response, Neuroinflammation, Serotonin, Neurogenesis and Synaptic Plasticity. Antioxidants.

[57] Cao L., Pi W., Zhang Y., Zheng C., Yong V.W., Xue M. (2025). Targeting the NLRP3-ROS Axis: Disrupting the Oxidative-Inflammatory Vicious Cycle in Intracerebral Hemorrhage. J. Inflamm. Res..

[58] Gasser P.J., Lowry C.A. (2018). Organic cation transporter 3: A cellular mechanism underlying rapid, non-genomic glucocorticoid regulation of monoaminergic neurotransmission, physiology, and behavior. Horm. Behav..

[59] Grunewald M., Johnson S., Lu D., Wang Z., Lomberk G., Albert P.R., Stockmeier C.A., Meyer J.H., Urrutia R., Miczek K.A. (2012). Mechanistic Role for a Novel Glucocorticoid-KLF11 (TIEG2) Protein Pathway in Stress-induced Monoamine Oxidase A Expression. J. Biol. Chem..

[60] Dhiman P., Malik N., Sobarzo-Sánchez E., Uriarte E., Khatkar A. (2019). Quercetin and Related Chromenone Derivatives as Monoamine Oxidase Inhibitors: Targeting Neurological and Mental Disorders. Molecules.

[61] Xiaopeng W., Zhan J., Fan Z., Chen Y., Jing W., Zhang M., Gan C., Yang J. (2024). Discovery of MAO-A Inhibitors as Antidepressant Based on Virtual Screening. Lett. Drug Des. Discov..

[62] Basak A.K., Chatterjee T. (2017). Pathogenesis of Major Depression by Stress Induced Hyperactivity of Hypothalmic-pituitary Adrenal Axis Mediated by Dysregulations of Neurotransmitter, Innate Immune Systems and Altered Brain micro RNA Expressions. Curr. Psychiatry Rev..

[63] Talarico F., Chakravarty S., Liu Y.S., Greenshaw A.J., Passos I.C., Cao B. (2023). Systematic Review of Psychiatric Adverse Effects Induced by Chloroquine and Hydroxychloroquine: Case Reports and Population Studies. Ann. Pharmacother..

[64] Roustit M., Guilhaumou R., Molimard M., Drici M.D., Laporte S., Montastruc J.-L. (2020). Chloroquine and hydroxychloroquine in the management of COVID-19: Much kerfuffle but little evidence. Therapies.

[65] Xu H., Zhang X.Y., Wang W.W., Wang J. (2021). Hydroxychloroquine Increased Anxiety-Like Behaviors and Disrupted the Expression of Some Related Genes in the Mouse Brain. Front. Pharmacol..

[66] Rapparini L., Cedirian S., La Placa M., Piraccini B.M., Raschi E., Starace M. (2025). Safety of Hydroxychloroquine: What a Dermatologist Should Know. Am. J. Clin. Dermatol..

[67] Mondal K., Porter H., Cole J., Pandya H.K., Basu S.K., Chiu C.Y., Shah V., Stephenson D.J., Chalfant C.E., Mandal N. (2022). Hydroxychloroquine Causes Early Inner Retinal Toxicity and Affects Autophagosome-Lysosomal Pathway and Sphingolipid Metabolism in the Retina. Mol. Neurobiol..

[68] Moawad P., Shamma R., Hassanein D., Ragab G., El Zawahry O. (2022). Evaluation of the effect of topical tacrolimus 0.03% versus cyclosporine 0.05% in the treatment of dry eye secondary to Sjogren syndrome. Eur. J. Ophthalmol..

[69] Yu J.-J., Zhang Y., Wang Y., Wen Z.-Y., Liu X.-H., Qin J., Yang J.-L. (2013). Inhibition of calcineurin in the prefrontal cortex induced depressive-like behavior through mTOR signaling pathway. Psychopharmacology.

[70] Zhu W., Shi H., Wang S., Wu P., Ding Z., Lu L. (2011). Hippocampal CA3 calcineurin activity participates in depressive-like behavior in rats. J. Neurochem..

[71] Seimandi M., Seyer P., Park C.S., Vandermoere F., Chanrion B., Bockaert J., Mansuy I.M., Marin P. (2013). Calcineurin interacts with the serotonin transporter c-terminus to modulate its plasma membrane expression and serotonin uptake. J. Neurosci..

[72] Dohgu S., Yamauchi A., Nakagawa S., Takata F., Kai M., Egawa T., Naito M., Tsuruo T., Sawada Y., Niwa M. (2004). Nitric oxide mediates cyclosporine-induced impairment of the blood–brain barrier in cocultures of mouse brain endothelial cells and rat astrocytes. Eur. J. Pharmacol..

[73] Cho H.J., Yoo J.J., Yun C.Y., Kang E.H., Lee H.-J., Hyon J.Y., Song Y.W., Lee Y.J. (2013). The EULAR Sjogren’s syndrome patient reported index as an independent determinant of health-related quality of life in primary Sjogren’s syndrome patients: In comparison with non-Sjogren’s sicca patients. Rheumatology.

[74] Bergdahl J., Bergdahl M. (2001). Environmental illness: Evaluation of salivary flow, symptoms, diseases, medications, and psychological factors. Acta Odontol. Scand..

[75] Milin M., Cornec D., Chastaing M., Griner V., Berrouiguet S., Nowak E., Marhadour T., Saraux A., Devauchelle-Pensec V. (2016). Sicca symptoms are associated with similar fatigue, anxiety, depression, and quality-of-life impairments in patients with and without primary Sjögren’s syndrome. Jt. Bone Spine.

[76] Rusthen S., Young A., Herlofson B.B., Aqrawi L.A., Rykke M., Hove L.H., Palm Ø., Jensen J.L., Singh P.B. (2017). Oral disorders, saliva secretion, and oral health-related quality of life in patients with primary Sjögren’s syndrome. Eur. J. Oral Sci..

[77] Gonzales J.A., Chou A., Rose-Nussbaumer J.R., Bunya V.Y., Criswell L.A., Shiboski C.H., Lietman T.M. (2018). How Are Ocular Signs and Symptoms of Dry Eye Associated with Depression in Women with and Without Sjögren Syndrome?. Am. J. Ophthalmol..

[78] Segal B., Bowman S.J., Fox P.C., Vivino F.B., Murukutla N., Brodscholl J., Ogale S., McLean L. (2009). Primary Sjögren’s Syndrome: Health experiences and predictors of health quality among patients in the United States. Health Qual. Life Outcomes.

[79] Chung S.W., Hur J., Ha Y.J., Kang E.H., Hyon J.Y., Lee H.J., Song Y.W., Lee Y.J. (2019). Impact of sleep quality on clinical features of primary Sjögren’s syndrome. Korean J. Intern. Med..

[80] Cui Y., Li L., Yin R., Zhao Q., Chen S., Zhang Q., Shen B. (2018). Depression in primary Sjögren’s syndrome: A systematic review and meta-analysis. Psychol. Health Med..

[81] Milic V., Grujic M., Barisic J., Marinkovic-Eric J., Duisin D., Cirkovic A., Damjanov N. (2019). Personality, depression and anxiety in primary Sjogren’s syndrome—Association with sociodemographic factors and comorbidity. PLoS ONE.

[82] Jabbi M., Arasappan D., Eickhoff S.B., Strakowski S.M., Nemeroff C.B., Hofmann H.A. (2020). Neuro-transcriptomic signatures for mood disorder morbidity and suicide mortality. J. Psychiatr. Res..

[83] Barnes J., Mondelli V., Pariante C.M. (2017). Genetic Contributions of Inflammation to Depression. Neuropsychopharmacology.

[84] Majidi J., Kosari-Nasab M., Salari A.-A. (2016). Developmental minocycline treatment reverses the effects of neonatal immune activation on anxiety- and depression-like behaviors, hippocampal inflammation, and HPA axis activity in adult mice. Brain Res. Bull..

[85] Giacobbo B.L., Doorduin J., Klein H.C., Dierckx R.A.J.O., Bromberg E., de Vries E.F.J. (2019). Brain-Derived Neurotrophic Factor in Brain Disorders: Focus on Neuroinflammation. Mol. Neurobiol..

[86] Zhu C.-B., Lindler K.M., Owens A.W., Daws L.C., Blakely R.D., Hewlett W.A. (2010). Interleukin-1 receptor activation by systemic lipopolysaccharide induces behavioral despair linked to MAPK regulation of CNS serotonin transporters. Neuropsychopharmacology.

[87] Felger J.C., Li L., Marvar P.J., Woolwine B.J., Harrison D.G., Raison C.L., Miller A.H. (2013). Tyrosine metabolism during interferon-alpha administration: Association with fatigue and CSF dopamine concentrations. Brain Behav. Immun..

[88] Bouças A.P., Rheinheimer J., Lagopoulos J. (2022). Why Severe COVID-19 Patients Are at Greater Risk of Developing Depression: A Molecular Perspective. Neuroscientist.

[89] D’Mello C., Le T., Swain M.G. (2009). Cerebral microglia recruit monocytes into the brain in response to tumor necrosis factoralpha signaling during peripheral organ inflammation. J. Neurosci..

[90] Turkheimer F.E., Veronese M., Mondelli V., Cash D., Pariante C.M. (2023). Sickness behaviour and depression: An updated model of peripheral-central immunity interactions. Brain Behav. Immun..

[91] Fasick V., Spengler R.N., Samankan S., Nader N.D., Ignatowski T.A. (2015). The hippocampus and TNF: Common links between chronic pain and depression. Neurosci. Biobehav. Rev..

[92] Dantzer R., O’Connor J.C., Freund G.G., Johnson R.W., Kelley K.W. (2008). From inflammation to sickness and depression: When the immune system subjugates the brain. Nat. Rev. Neurosci..

[93] Banks W.A., Kastin A.J., Broadwell R.D. (1995). Passage of cytokines across the blood-brain barrier. Neuroimmunomodulation.

[94] Morita S., Furube E., Mannari T., Okuda H., Tatsumi K., Wanaka A., Miyata S. (2016). Heterogeneous vascular permeability and alternative diffusion barrier in sensory circumventricular organs of adult mouse brain. Cell Tissue Res..

[95] Miyata S. (2015). New aspects in fenestrated capillary and tissue dynamics in the sensory circumventricular organs of adult brains. Front. Neurosci..

[96] Liu Y.-D., Chang Y.-H., Xie X.-T., Wang X.-Y., Ma H.-Y., Liu M.-C., Zhang H.-M. (2025). PET Imaging Unveils Neuroinflammatory Mechanisms in Psychiatric Disorders: From Microglial Activation to Therapeutic Innovation. Mol. Neurobiol..

[97] Singhal G., Singhal S., Baune B.T. (2025). Role of astrocyte in neuroinflammation-induced loss in neuroplasticity and subsequent onset of depression: A systematic review. Neuroprotection.

[98] Bose M.M., Govindula A., Nampoothiri M., Arora D., Mudgal J. (2026). The HPA axis and kynurenine pathway: Exploring the role of stress and neuroinflammation in treatment-resistant depression. Pharmacol. Rep..

[99] Ma H., Li C., Wang J., Zhang X., Li M., Zhang R., Huang Z., Zhang Y. (2021). Amygdala-hippocampal innervation modulates stress-induced depressive-like behaviors through AMPA receptors. Proc. Natl. Acad. Sci. USA.

[100] Peng G.-J., Tian J.-S., Gao X.-X., Zhou Y.-Z., Qin X.-M. (2015). Research on the Pathological Mechanism and Drug Treatment Mechanism of Depression. Curr. Neuropharmacol..

[101] Li X.X., Maitiyaer M., Tan Q., Huang W.H., Liu Y., Liu Z.P., Wen Y.Q., Zheng Y., Chen X., Chen R.L. (2024). Emerging biologic frontiers for Sjogren’s syndrome: Unveiling novel approaches with emphasis on extra glandular pathology. Front. Pharmacol..

[102] Carding S., Verbeke K., Vipond D.T., Corfe B.M., Owen L.J. (2015). Dysbiosis of the gut microbiota in disease. Microb. Ecol. Health Dis..

[103] De Paiva C.S., Jones D.B., Stern M.E., Bian F., Moore Q.L., Corbiere S., Streckfus C.F., Hutchinson D.S., Ajami N.J., Pe-trosino J.F. (2016). Altered Mucosal Microbiome Diversity and Disease Severity in Sjögren Syndrome. Sci. Rep..

[104] Yang L., Xiang Z., Zou J., Zhang Y., Ni Y., Yang J. (2022). Comprehensive Analysis of the Relationships Between the Gut Microbiota and Fecal Metabolome in Individuals with Primary Sjogren’s Syndrome by 16S rRNA Sequencing and LC–MS-Based Metabolomics. Front. Immunol..

[105] Moon J., Choi S.H., Yoon C.H., Kim M.K. (2020). Gut dysbiosis is prevailing in Sjögren’s syndrome and is related to dry eye severity. PLoS ONE.

[106] Wang X., Wang J., Guo W., Zhou Y., Sun C., Li Z., Chen L., Pan X. (2020). Characteristics of intestinal flora in patients with primary Sjögren syndrome. J. South Med. Univ..

[107] Yuan X., Wang J., Wang W., Song Y., Wu J., Du R. (2025). Microbiome alterations in primary Sjögren’s syndrome: Regional dysbiosis and microbiome-targeted therapeutic strategies. Clin. Immunol..

[108] Kuklinski E., Asbell P.A. (2017). Sjogren’s syndrome from the perspective of ophthalmology. Clin. Immunol..

[109] Zilliox M.J., Gange W.S., Kuffel G., Mores C.R., Joyce C., de Bustros P., Bouchard C.S. (2020). Assessing the ocular surface microbiome in severe ocular surface diseases. Ocul. Surf..

[110] Schaefer L., Trujillo-Vargas C.M., Midani F.S., Pflugfelder S.C., Britton R.A., de Paiva C.S. (2022). Gut Microbiota From Sjögren syndrome Patients Causes Decreased T Regulatory Cells in the Lymphoid Organs and Desiccation-Induced Corneal Barrier Disruption in Mice. Front. Med..

[111] Catena-Dell’OSso M., Bellantuono C., Consoli G., Baroni S., Rotella F., Marazziti D. (2011). Inflammatory and neurodegenerative pathways in depression: A new avenue for antidepressant development?. Curr. Med. Chem..

[112] Eyre H., Baune B.T. (2012). Neuroplastic changes in depression: A role for the immune system. Psychoneuroendocrinology.

[113] Mhanna A., Martini N., Hmaydoosh G., Hamwi G., Jarjanazi M., Zaifah G., Kazzazo R., Mohamad A.H., Alshehabi Z. (2024). The correlation between gut microbiota and both neurotransmitters and mental disorders: A narrative review. Medicine.

[114] Mccarron R.M., Shapiro B., Rawles J., Luo J. (2021). Depression. Ann. Intern. Med..

[115] Alexander E.L., Beall S.S., Gordon B., Selnes O.A., Yannakakis G.D., Patronas N., Provost T.T., McFarland H.F. (1988). Magnetic resonance imaging of cerebral lesions in patients with the Sjögren syndrome. Ann. Intern. Med..

[116] Chaudhury D., Walsh J.J., Friedman A.K., Juarez B., Ku S.M., Koo J.W., Ferguson D., Tsai H.-C., Pomeranz L., Christoffel D.J. (2013). Rapid regulation of depression-related behaviours by control of midbrain dopamine neurons. Nature.

[117] Sharma A., Dhooria A., Aggarwal A., Rathi M., Chandran V. (2016). Connective Tissue Disorder-Associated Vasculitis. Curr. Rheumatol. Rep..

[118] Akasbi M., Berenguer J., Saiz A., Brito-Zerón P., Pérez-De-Lis M., Bové A., Diaz-Lagares C., Retamozo S., Blanco Y., Perez-Alvarez R. (2012). White matter abnormalities in primary Sjögren syndrome. QJM.

[119] Andrianopoulou A., Zikou A.K., Astrakas L.G., Gerolymatou N., Xydis V., Voulgari P., Kiortsis D.-N., Argyropoulou M.I. (2020). Functional connectivity and microstructural changes of the brain in primary Sjögren syndrome: The relationship with depression. Acta Radiol..

[120] Lauvsnes M.B., Maroni S.S., Appenzeller S., Beyer M.K., Greve O.J., Kvaløy J.T., Kvaløy J.T., Gøransson L.G., Tjensvoll A.B., Omdal R. (2013). Memory dysfunction in primary Sjögren’s syndrome is associated with anti-NR2 antibodies. Arthritis Rheum..

[121] Lauvsnes M.B., Beyer M.K., Kvaløy J.T., Greve O.J., Appenzeller S., Kvivik I., Harboe E., Tjensvoll A.B., Gøransson L.G., Omdal R. (2014). Association of hippocampal atrophy with cerebrospinal fluid antibodies against the NR2 subtype of the N-methyl-D-aspartate receptor in patients with systemic lupus erythematosus and patients with primary Sjögren’s syndrome. Arthritis Rheumatol..

[122] Hamieh A., Mallaret G., Meleine M., Lashermes A., Roumeau S., Boudieu L., Barbier J., Aissouni Y., Ardid D., Gewirtz A. (2021). Toll-like receptor 5 knock-out mice exhibit a specific low level of anxiety. Brain Behav. Immun..

[123] Thalayasingam N., Baldwin K., Judd C., Ng W.-F. (2021). New developments in Sjogren’s syndrome. Rheumatology.

[124] Khan A., Shirlaw P.J. (2016). Dry Mouth and Sjögren’s Syndrome: An Overview. Prim. Dent. J..

[125] Barsottini O.G.P., de Moraes M.P.M., Fraiman P.H.A., Marussi V.H.R., de Souza A.W.S., Neto P.B., Spitz M. (2023). Sjogren’s syndrome: A neurological perspective. Arq. Neuro-Psiquiatr..

[126] Westhoff G., Dörner T., Zink A. (2012). Fatigue and depression predict physician visits and work disability in women with primary Sjögren’s syndrome: Results from a cohort study. Rheumatology.

[127] Holzchuh R., Villa Albers M.B., Osaki T.H., Igami T.Z., Santo R.M., Kara-Jose N., Holzchuh N., Hida R.Y. (2011). Two-year outcome of partial lacrimal punctal occlusion in the management of dry eye related to Sjögren syndrome. Curr. Eye Res..

[128] Brito-Zerón P., Retamozo S., Ramos-Casals M. (2023). Sjögren syndrome. Med. Clin..

[129] Liu Z., Dong Z., Liang X., Liu J., Xuan L., Wang J., Zhang G., Zhang G., Hao W. (2017). Health-related quality of life and psy-chological status of women with primary Sjögren’s syndrome: A cross-sectional study of 304 Chinese patients. Medicine.

[130] Miyamoto S.T., Valim V., Carletti L., Ng W.-F., Perez A.J., Lendrem D.W., Trennel M., Giovelli R.A., Dias L.H., Serrano É.V. (2019). Supervised walking improves cardiorespiratory fitness, exercise tolerance, and fatigue in women with primary Sjögren’s syndrome: A randomized-controlled trial. Rheumatol. Int..

